# Supporting students from underrepresented minority backgrounds in graduate school: A mixed-methods formative study to inform post-baccalaureate design

**DOI:** 10.1017/cts.2024.590

**Published:** 2024-09-16

**Authors:** Jessica B. Sperling, Noelle E. Wyman Roth, Whitney E. Welsh, Allison T. McElvaine, Sallie R. Permar, Rasheed A. Gbadegesin

**Affiliations:** 1 Duke University Social Science Research Institute, Durham, NC, USA; 2 Duke University Clinical and Translational Science Institute, Durham, NC, USA; 3 Duke University School of Medicine, Durham, NC, USA; 4 Weill Cornell Medicine, New York, NY, USA

**Keywords:** Pipeline development programs, underrepresented minorities, diversity, evaluation, recruitment and retention

## Abstract

The US biomedical research workforce suffers from systemic barriers causing insufficient diversity and perpetuating inequity. To inform programming enhancing graduate program access, we implemented a formative mixed-method study to identify needed supports for program applications and graduate program success. Overall, results indicate value in added supports for understanding application needs, network development, critical thinking, time management, and reading academic/scientific literature. We find selected differences for underrepresented minority (URM) students compared to others, including in the value of psychosocial supports. This work can inform broader efforts to enhance graduate school access and provides foundation for further understanding of URM students’ experiences.

The USA faces a biomedical workforce crisis, specifically in research; 30 years ago, 4% of individuals in the biomedical workforce identified as researchers, and now that number is just 1% [1]. In addition, this constrained workforce is characterized by insufficient recruitment and retention of individuals from underrepresented backgrounds in biomedical training [2]. Among those initially recruited into science, technology, engineering, and mathematics (STEM) education, attrition from these fields starts at the beginning of undergraduate training, and students from underrepresented racial and ethnic group (also referred to as underrepresented minority, or URM) backgrounds exit STEM majors at an especially high rate, and disproportionate attrition perpetuates through training, career, and leadership positions. While racial and ethnic minorities comprise one-third of the US population, they represent only 10% of life science PhDs – and just 5% of tenure-track faculty in the biological sciences [3]. Systemic and structural barriers causing insufficient diversity, maintaining inequity, and limiting inclusion lead to the perpetuation of inequities in both research and healthcare delivery [1].

With support from the Burroughs Wellcome Fund, [blinded Entity 1] [4–6] launched an initiative to support individuals from URM backgrounds who have biomedical career interests. It developed a post-baccalaureate program to prepare students interested in pursuing a biomedical research career for acceptance to, and success in, graduate biomedical or medical scientist programs (MD or MD/PhD). Prior to program launch, [blinded Entity 1] partnered with [blinded Entity 2] to conduct a formative evaluation research study to determine areas of need among undergraduates and recent graduates at diverse academic institutions who are interested in biomedical careers. This aimed to inform the developing [blinded primary program name] program and other initiatives working to prepare individuals for successful completion of biomedical degree programs and to generate a cohort of diverse, well-trained scientists to strengthen the research enterprise.

## Method

This project was guided by the following questions: What are the current challenges facing undergraduate students in gaining admission to and succeeding in biomedical graduate school? What supports or infrastructures may facilitate admission to and success in medical or graduate school? What factors are particularly salient for individuals from URM backgrounds?

An exploratory sequential mixed-method empirical process addressed these questions [7]. Phase 1 included qualitative data collection with key informants to help inform factors for success and key challenges that would be addressed in a Phase 2 graduate student survey. This included focus groups with 10 faculty members from the [blinded Regional STEM Diversity Coalition] and thematic analysis of focus group data using NVivo 12. More information about Phase 1 methodology, including the focus group guide, is available as Supplemental Digital Appendix 1. Results from these focus groups informed the content and constructs included in a subsequent Phase 2 graduate student survey. The survey addressed areas including: skills and experiences needed for application to/success in a biomedical graduate degree, mentorship experience, and psychosocial challenges. Respondents were primarily recruited through [blinded Primary University] and secondarily via contacts at other institutions [blinded Universities 2–4] and [blinded Regional STEM Diversity Coalition] members; Table 1 shows participant race/ethnicity, gender, and URM status. Quantitative data were analyzed in SAS 9.4 and included descriptive statistics, Cohen’s *d* statistics to measure effect size of difference, independent t-tests to examine group differences, and selected ordinary least squares (OLS) regressions. Open-ended survey data were analyzed for themes using NVivo 12 [8]. More information on Phase 2 methodology and instruments is in Appendix 2.

**Table 1.** Survey respondent demographics (*n* = 244)*

Category	Group/demographic	Number	Percent
Race/ethnicity	Asian	72	29.5%
	Black	12	4.9%
	Latino	30	12.3%
	Native	0	0.0%
	Hawaiian	0	0.0%
	Pacific Islander	1	0.4%
	Middle Eastern	4	1.6%
	White	152	62.3%
Gender identity	Female	163	67%
	Male	71	29%
	Non-binary/third gender	7	3%
	Self-identify	1	0%
	Prefer not to say	2	1%
NIH-designated underrepresented minority (URM)	URM	39	16.0%
	Non-URM	205	84.0%

* *N* = 3 respondents did not respond to demographic questions and are excluded from this table, with total respondents including those missing demographic data = 247. These three respondents were not included in any analyses including demographic data (i.e., URM and non-URM-specific analyses).

Duke University Campus IRB approval was waived for key informant focus groups (IRB determined faculty involvement as expert consultation; 2/21/2021) and was granted for survey data (2022-0060, approved 10/8/2021).

## Results

### Phase 1: key informant faculty focus groups

Respondents reported that students’ early consideration of biomedical research careers, critical preparatory coursework, and research experience were key facilitators of biomedical graduate program entry. Additional critical skills and experiences included GRE/MCAT preparation, reading primary (academic or scientific) literature, basic laboratory competency, quantitative proficiency, critical thinking and analytic skills, writing/communication skills, and time management. Respondents reported that lack of awareness of biological science opportunities outside of medicine can be more prominent for URMs, first generation, and/or those attending non-R1 institutions. This lack of awareness was rooted in multiple factors including lack of role models. In addition, students at smaller or non-R1 undergraduate institutions may lack sufficient research experience, both in terms of quantity and quality, to be competitive graduate school candidates.

Respondents also noted challenges URM-identified students can face based on differences in their approach and confidence. For instance, attending an undergraduate institution with high admission rates, and high performance relative to their peers at their undergraduate institution, can lead to a less competitive mindset that can affect their likelihood of pursuing opportunities to improve chances of graduate school admission and, if admitted, preparation for rigor and competitiveness of study. Respondents reported that some URMs, especially Black or first-generation immigrants, may come from cultures that discourage assertiveness, leading them to be less likely to propose or advocate for their own research agendas than peers. Respondents indicated how imposter syndrome and can be exacerbated by a lack of diversity in a program and other direct experiences of racism or sexism.

Respondents addressed the deliberate cultivation of supportive communities a defining feature of successful training and preparatory programs and particularly consequential for underrepresented racial/ethnic minorities and women. Mentorship was also seen as essential and extending beyond faculty and principal investigators, needing to encompass post-docs and graduate students with more day-to-day involvement with students. Supportive institutional culture, especially in the laboratory, and strong mentorship (or the lack thereof) was often described as the determining factor in post-baccalaureate and first-year graduate students’ decisions of whether to continue in the biological sciences.

### Phase 2: graduate student survey

#### Application to graduate programs

Survey respondents indicated that all skills/experienced assessed, conceptualized as potentially important to a successful biomedical degree program applications, were indeed at least moderately important (Table 2). Knowledge of how to complete applications and having good recommendation letters were rated as most essential. URM respondents indicated that time management and recommendations were significantly more important compared to non-URM respondents. In ratings of respondents’ strength at the time of their application to their degree program, URM respondents reported lower skills in basic laboratory competency compared to their non-URM peers (approaching significance, p < 0.10); however, URM respondents reported feeling marginally stronger than non-URM respondents in ability to complete applications and significantly stronger in good recommendation letters. Of note, focus group key informants indicated that URM participants, depending on their undergraduate institution, may not always realizing how strong their competition is for graduate school admission; this may be related to these sentiments of greater strength in these areas. Importance-strength discrepancies overall (i.e., the difference in means between relative importance and strength when applying) were greatest for in network connections and knowing what is needed for an application; URM respondents indicated the greatest discrepancies in network connections and as well as research/lab experience.

**Table 2.** Importance of specific factors and strength in factors when applying to postgraduate institution (*n* = 241)^a^

Skill/experience/factor	Overall				URM^b^				Non-URM^c^			
	Importance to application^d^		Strength when applying^e^		Importance to application		Strength when applying		Importance to application		Strength when applying	
	Mean	*SD*	Mean	*SD*	Mean	*SD*	Mean	*SD*	Mean	*SD*	Mean	*SD*
*Research specific skill/experience*												
Research/lab experience	6.43	*0.75*	5.81	*1.18*	6.38	*0.75*	5.61	*1.41*	6.44	*0.76*	5.84	*1.14*
Reading academic/scientific literature	4.69	*1.41*	4.65	*1.38*	4.79	*1.49*	4.87	*1.65*	4.66	*1.4*	4.6	*1.32*
Basic laboratory competency	5.15	*1.54*	5.58	*1.37*	5.28	*1.56*	5.24	*1.51*	5.12	*1.54*	5.64	*1.35*
Quantitative skills	4.37	*1.29*	4.59	*1.41*	4.62	*1.25*	4.61	*1.42*	4.32	*1.3*	4.58	*1.42*
Critical thinking	6.25	*0.88*	5.67	*0.95*	6.15	*0.9*	5.61	*1.15*	6.27	*0.88*	5.67	*0.91*
*Professional competency/”soft” skill*												
Writing and communication skills	6.24	*0.82*	5.6	*1.08*	6.31	*0.95*	5.68	*1.07*	6.24	*0.79*	5.58	*1.09*
Time management	5.78	*1.14*	5.37	*1.29*	6.13	*0.86*	5.58	*1.27*	5.72	*1.18*	5.32	*1.3*
Network connections	5.26	*1.28*	4.37	*1.61*	5.38	*1.29*	4.43	*1.59*	5.23	*1.28*	4.35	*1.61*
*Application skill/experience*												
Knowing what is needed for an application	6.53	*1.03*	5.72	*1.23*	6.59	*1.04*	5.95	*1.21*	6.52	*1.04*	5.67	*1.24*
Good recommendation letter(s)	6.54	*0.71*	6	*1.02*	6.85	*0.37*	6.39	*0.79*	6.48	*0.75*	5.93	*1.04*
Completing applications	6.81	*0.72*	6.25	*1.01*	6.92	*0.35*	6.53	*0.86*	6.8	*0.77*	6.2	*1.03*
GRE/MCAT preparation	4.64	*1.97*	5.02	*1.59*	4.36	*2.21*	4.78	*1.51*	4.67	*1.93*	5.04	*1.61*

a N for overall participants ranged from 240 to 241. Importance scale is 1–7, where 1 = not at all important and 7 = absolutely critical. Strength scale is 1–7, where 1 = extremely weak [novice] and 7 = extremely strong [expert].

b N for underrepresented minority (URM) participants ranged from 37 to 39.

c N for non-URM participants ranged from 200 to 205.

#### Success in graduate programs

Respondents considered all the skills and experiences indicated in the survey to be at least moderately important to success in their degree program, with critical thinking and time management as most important; see Table 3. However, URM respondents rated most skills and experiences as more important to success than non-URM respondents. In addition, as with the application itself, URM respondents stated that time management was more important compared to non-URM respondents (approaching significance, *p* < 0.10). Among research and professional competency skills directly assessed, reading scientific/academic literature, quantitative skills, network connections, and time management were described as most challenging for respondents. URM respondents indicated that network connections were significantly less challenging than non-URM respondents. Beyond skills, respondents reported that mentorship was a valuable support. Though the difference was not statistically significant, URM respondents reported that mentorship was more helpful compared to non-URM respondents. In an open-ended question about advice for incoming students (*n* = 202 responses), mentorship was second most frequently coded theme, and proportionately more URM respondents provided advice on the value of mentorship compared to non-URM respondents. Respondents spoke to the value of multiple mentorships, including developing relationships with faculty other than one’s advisor and seeking advice from peers.

**Table 3.** Importance of specific skills/experiences to success compared to challenge in graduate program (*n* = 245)^a^

Skill/experience	Overall				URM^b^				Non-URM^c^			
	Importance to success		Challenge		Importance to success		Challenge		Importance to success		Challenge	
	Mean	*SD*	Mean	*SD*	Mean	*SD*	Mean	*SD*	Mean	*SD*	Mean	*SD*
*Research-specific skill/experience*												
Research/lab experience	6.15	*1.15*	3.83	*1.58*	6.23	*1.25*	3.54	*1.76*	6.12	*1.13*	3.9	*1.54*
Reading academic/scientific literature	5.98	*1.28*	3.36	*1.45*	6.18	*1.21*	3.28	*1.56*	5.94	*1.3*	3.38	*1.44*
Basic laboratory competency	5.51	*1.73*	4.82	*1.54*	5.69	*1.66*	4.69	*1.75*	5.49	*1.75*	4.87	*1.49*
Quantitative skills	5.04	*1.39*	3.56	*1.45*	5.26	*1.33*	3.59	*1.37*	5	*1.41*	3.53	*1.46*
Critical thinking	6.58	*0.84*	3.83	*1.54*	6.56	*0.68*	4.1	*1.54*	6.58	*0.88*	3.76	*1.54*
*Professional competency/“soft”skill*												
Writing and communication skills	6.14	*1.13*	3.94	*1.67*	6.18	*1.17*	4.23	*1.81*	6.14	*1.13*	3.87	*1.65*
Time management	6.37	*0.93*	3.74	*1.64*	6.56	*0.64*	3.77	*1.74*	6.33	*0.97*	3.72	*1.63*
Network connections	5.53	*1.24*	3.66	*1.65*	5.74	*1.21*	4.36	*1.86*	5.5	*1.24*	3.55	*1.57*

a *N* ranged from 242 to 245 overall. Importance to success of key factors uses scale 1–7, where 1 = not at all important and 7 = absolutely critical. Challenge in graduate program uses the scale 1–7, where 1 = extremely challenging and 7 = extremely easy.

b *N* = 39 for underrepresented minority (URM) respondents.

c *N* ranged from 200 to 203 for non-URM respondents.

Among all respondents, there were the greatest importance/challenge gap (importance for success vs. challenge experienced) for critical thinking, time management, and reading academic/scientific literature. URM respondents indicated the greatest gaps in reading academic/scientific literature, time management, and research/lab experience.

Regarding psychosocial and cultural challenges, respondents overall rated imposter syndrome (i.e., feelings of inadequacy) as the most challenging, followed by feelings of isolation. Respondents from URM backgrounds reported imposter syndrome as significantly more challenging compared to non-URM respondents and reported the competitive culture of their graduate program and feelings of isolation as marginally more challenging (*p* < .10). Overall, 42% of qualitative responses on advice to incoming students addressed psychosocial factors, representing the most frequently coded theme. URM respondents addressed psychosocial challenges at similar rates as non-URM respondents, but the content of that advice varied. Proportionately more URM respondents addressed finding a supportive community, self-care and wellbeing, and advice related to imposter syndrome; proportionately more non-URM respondents emphasized the importance of asking for help.

## Discussion & conclusion

As skills assessed in the survey were considered at least slightly important to success during their graduate degree, they should be considered for post-baccalaureate programming. Post-baccalaureate programming could pay particular attention to skills and experiences where students face the greatest challenge or need relative to this skill/experience’s importance. For admission to graduate school, this includes knowing what is needed for an application and network connections; for success in graduate school, this includes critical thinking, time management, and ability to read academic/scientific literature. There are numerous areas in which we do not find significant differences between URM and non-URM students. This is critical to consider as it indicates that results may apply across students of different backgrounds and thus should be applied to any support programming. Yet, analyses speak to value of specific supports for URM-identifying students in select areas including research/lab experience, basic laboratory competencies, reading academic/scientific literature, and time management. URM respondents also reported greater psychosocial and cultural challenges, which suggests post-baccalaureate programs should consider how to best support and prepare these students for those challenges, particularly given historical institutional and structural sexism and racism within education, including within STEM higher education, that has systematically excluded women racial and ethnic minorities and led to specific challenges including lowered sense of belonging and self-efficacy [9–12]. Mentorship emerged as highly important, and URM respondents indicated relatively higher value for mentorship as a support compared to their peers; this echoes other work emphasizing the value of mentorship [13,14]. Mentorships established during post-baccalaureate can offer important support throughout graduate training, and a post-baccalaureate program could prepare students with an understanding of the importance of or strategies for identifying mentors in graduate school.

For [blinded primary program], this formative evaluation informed recruitment, application and review criteria, and mentor matching, and [blinded primary program] implemented recommendations from the study (e.g., providing specific support for desired skills, connecting participants to another on-campus program with an additional cohort of students, and strong mentorship). This formative evaluation has also informed efforts beyond [blinded primary program], such as the separate [blinded program 2] and can inform other related programs’ development, as well as broader research on support for biomedical graduate program success in general and for URM students.

Yet, this study, while contributing to knowledge on supporting a diverse biomedical field, has select limitations. The survey phase drew primarily from respondent trainees at one specific university, and it is possible that experience varies by institution. A larger sample size, particularly of URM respondents, may have highlighted more and/or varied statistically significant differences. Added research could further explore psychosocial and cultural challenges, including qualitative data collection (e.g., interviews and focus groups). Additional research could also further explore the role of undergraduate institution type, including designation (HBCU, R1, etc.) and students’ experience (e.g., degree of prior competitive culture) in informing their experience applying to and during graduate school. These research directions, building on the preliminary work discussed here, could further inform programs supporting biomedical graduate students.

## Supporting information

Sperling et al. supplementary material Sperling et al. supplementary material

## Supplementary material

The supplementary material for this article can be found at http://dx.doi.org/10.1017/cts.2024.590.
