# Supporting students from underrepresented minority backgrounds in graduate school: A mixed-methods formative study to inform post-baccalaureate design – ADDENDUM

**DOI:** 10.1017/cts.2025.10150

**Published:** 2025-11-27

**Authors:** Jessica B. Sperling, Noelle E. Wyman Roth, Whitney E. Welsh, Allison T. McElvaine, Sallie R. Permar, Rasheed A. Gbadegesin

An addition has been made to the acknowledgements section of this article. Please see the full acknowledgements section below.

(*)Acknowledgments: The authors wish to thank the respondents who contributed to the data upon which this manuscript is based. Doreet Preiss, PhD contributed to elements of methodology, analysis, and interpretation and review for an initial survey report that informed this publication. Stephanie Rosner, BA, as a graduate student intern, additionally contributed in initial steps of drawing together Phase 1 and Phase 2 results reports.
