# Supplementary material for: Supporting students from underrepresented minority backgrounds in graduate school: A mixed-methods formative study to inform post-baccalaureate design
Source: J Clin Transl Sci. 2024 Sep 16;8(1):e124. doi: 10.1017/cts.2024.590 (PMC11428063; doi:10.1017/cts.2024.590)
Supplement: Sperling et al. supplementary material [file S2059866124005909sup001.docx]

**Supplemental Digital Appendix**

**Appendix 1: Phase I Methodology**

**Participants, Recruitment, and Analysis**

Faculty respondents were included based on their ability to reflect upon a broad student population and across years of experience, with this group comprising STEM-focused leaders from representatives of 12 undergraduate institutions. This Coalition specifically was utilized based its aims, members’ roles related to [blinded primary program name] and institution types represented. [blinded Regional STEM Diversity Coalition] aims to develop and implement quality opportunities across the coalition that will prepare promising students from diverse backgrounds for success in pursuing careers as independent biomedical scientists and physician-scientists. It includes individuals who were intended to be key partners in development of the [blinded primary program name] program, already had some degree of knowledge of [blinded primary program name] intents, and had indicated willingness to inform this process. The Coalition included faculty who had significant experience with URM students, and it included representation of HBCUs and SLACs which permitted attention to these unique undergraduate institution circumstances and augmented [blinded primary institution’s] lens as a non-HBCU/non-SLAC. Focus groups addressed student access to research experiences, programs to prepare students for competitive graduate and medical school application, and resources for faculty.

All [blinded Regional STEM Diversity Coalition] faculty (n=12) were invited to participate, with 10 respondents participating in three focus groups in Spring 2021 (83% participation rate). Respondents represented six HBCUs, one of which was a SLAC, and three additional non-HBCU SLACs. We examined transcripts using inductive thematic analysis and NVivo 12 qualitative data analysis software. Focus group results highlighted key areas of need for preparation in graduate study and aspects of a graduate program that may facilitate success, including areas that may be particularly salient to students of minoritized ethnic/racial background; we ensured that survey items addressed these specific areas.

**Focus Group Data Collection Instrument**

*[developed as semi-structured focus group format]*

Introductions

We’d like to begin with introductions, so that we are all familiar with each other. Please introduce yourself by stating your name and organization affiliation (if you have multiple affiliations, you can include whatever is most relevant to your participation in this discussion).

Great, thanks! For today’s discussion, we are first going to consider what challenges students face when they are trying to gain admission to a biomedical graduate program – either PhD programs, or MD / PhD programs - and what programs or supports could help them meet those challenges. Then later in the discussion, we’ll talk about what challenges they face when it comes to succeeding in graduate school, and what programs or supports could better prepare them to meet those challenges.

While we talk, I’d like to know if any of your thoughts are different for a PhD program versus and MD/PhD program. So, if you are sharing thoughts that you think are salient for one but not the others, certainly note that.

Getting accepted to a biomedical graduate program

- I want to start with a big, but critical, question. Per your lens and experience, what is the most significant challenge or barrier your students face to getting into a graduate program (PhD or MD-PhD) in the biomedical sciences? Why?

(areas that should be top priority for PRIME)

*probe for: academics, GRE/MCAT prep, hard skills/scientific proficiency, research opportunities, soft skills/communication/writing/interviewing, networking/mentorship, flow of information about such programs, funding, application process*

- - *prompt*: Are there any other challenges or barriers to getting into a biomedical graduate program that have not yet been mentioned?
- Do you view any of these challenges as particularly pronounced for certain types of students? This could be students from of university contexts, certain majors, demographic groups, socioeconomic backgrounds, or anything else you see as salient. Why?

*probe for: race/ethnicity, disadvantaged socioeconomic background, disabilities; gender as a secondary concern*

- I’d like you to think about any other programs or supports do you know of, either at your institution or based elsewhere, to help students address gain entry into PhD or MD-PhD programs. Do these programs address the challenges we’ve discussed? How so /why not?
  - *prompt*: What, if anything, about them is effective? What key areas does the program not address? What would you change about the program to address this gap?

*probe for: description, effectiveness, timing – undergrad vs. post-bac;*

*training programs, research opportunities, networking, faculty resources*

- - Are these supports particularly valuable for certain types of students? Why?

*probe for: race/ethnicity, disadvantaged socioeconomic background, disabilities; gender as a secondary concern*

- - Are there particular challenges in these programs – either in access to them, or success in them - for certain types of students? How / why?

*probe for: race/ethnicity, disadvantaged socioeconomic background, disabilities; gender as a secondary concern – especially any mentioned above*

- - *[if PREP was not discussed above]* We know of PREP as a program to support admission to PhD programs. Is anyone here familiar with PREP?
  - What are some key areas that the PREP program does not address? What would you change about the program to address this gap?
- What are some areas where most students do not need help, or need limited help?

Succeeding in a biomedical graduate program

Okay, great! Thanks! Now we would like to discuss what challenges students face *after they’re enrolled* in a biomedical graduate program (PhD, or MD/PhD), and what programs or supports could better prepare them to meet those challenges. This is understanding that that what it takes to get admitted may be different from what it takes to succeed once you’re there.

As a note, we understand that you work most closely with undergraduates. If you don’t feel you have enough information to address success in graduate school, though, that is perfectly fine – you can just say so.

- What is the most significant challenge or barrier your students face when it comes to succeeding in a graduate program (PhD or MD-PhD) in the biomedical sciences?

(areas that should be top priority for PRIME)

*probe for: academic readiness, research experience, hard skills, soft skills (communication, networking, time management, self-motivation), transition from small to large school, funding*

- - *prompt*: Are there any other challenges or barriers to succeeding in a biomedical graduate program that have not yet been mentioned?
- Are these challenges that most students have, or are they more specific to certain types of students? For instance, students from certain types of university contexts, certain majors, demographic groups, socioeconomic backgrounds, or anything else you see as salient?

*probe for: race/ethnicity, disadvantaged socioeconomic background, disabilities; gender as a secondary concern*

- Are you familiar with any programs or supports to help students prepare for these challenges, either at your institution or elsewhere?

*probe for: description, effectiveness, timing – undergrad vs. post-bac*

- - *prompt*: Could you describe what about them is effective? Are there key areas that the program does not address? What would you change about the program to address this gap?
  - *prompt*: Are these supports particularly valuable for certain types of students?

*probe for: race/ethnicity, disadvantaged socioeconomic background, disabilities; gender as a secondary concern*

- Are there any other supports or programs that you think could help students better prepare for graduate programs in the biomedical sciences?

*probe for: whether programs should be targeted at certain types of students, especially around race/ethnicity, disadvantaged socioeconomic background, disabilities*

- What are some areas where most students do not need help, or need limited help?

Underrepresentation in biomedical graduate programs

Okay, great! Thanks! Finally, we would like to end with a bigger-picture lens.

- Aside from the specific supports we’ve discussed today, what else, if anything, do you think can be done to increase diversity in biomedical graduate programs?

Thank you so much for your time!

**Appendix 2. Phase 2 (Survey) Methodology**

**Participants, Recruitment, and Analysis**

[blinded Primary University] biomedical PhD and MD students were recruited through a distribution list provided by the School of Medicine, as a primary sample. An anonymous link was also shared through a [Primary University] postdoctoral associate newsletter and with faculty/staff contacts at other institutions ([blinded Universities 2-4], and via [blinded Regional STEM Diversity Coalition] members). We collected survey data through an online survey platform (Qualtrics) in early October 2021, with 247 respondents. For primary [blinded Primary University] respondents, who received a targeted individual emails (n=1089), 19.6% responded (n=213); the remaining 34 were from other institutions. Respondents from other institutions did not receive targeted emails, so response rates are unknown.

Quantitative were analyzed in SAS 9.4 and included descriptive statistics (e.g., means, standard deviations, frequencies), independent t-tests to examine mean group differences, and Cohen’s d statistics to measure effect size of difference. We examined independent items instead of creating a summary scale given the intent to examine specific aspects of preparation and experience. OLS regressions were additionally utilized when descriptive statistics or t-tests highlighted findings the team wanted to explore in more depth or when conceptual questions emerged that aligned with the research questions. We analyzed qualitative survey data using NVivo 12 with a focus on identifying key themes across respondents.8

In use of multiple t-tests, we determined not to include Bonferroni corrections; given this work as exploratory, and also accounting or the relatively smaller size of our URM subgroup, we wanted to minimize failure to detect differences that may be present.^10^ Using descriptive results, we also examined the discrepancies between a) relative importance of specific skills and experiences to a successful application and b) respondents’ strength when applying by subgroups (i.e., the difference in means between relative importance and strength when applying). This prioritized a focus on URM respondents compared to non-URM respondents, using NIH definitions of URM. In quantitative results including tests for statistical significance, we include results approaching significance (*p* between 0.1 and 0.05) given the exploratory nature of this work and the small sample size for some groups.

**Survey Data Collection Instrument**

*Note: The survey was programmed in Qualtrics, and the instrument below reflects this Qualtrics programming structure.*

Survey Flow

Standard: Background (8 Questions)

Standard: Experience in UG/Preparation for admission (6 Questions)

Standard: Application (6 Questions)

Branch: New Branch

If

If Did you complete a post-baccalaureate program? Yes Is Selected

Standard: Postbac (7 Questions)

Standard: Experience & needs in grad program (7 Questions)

Standard: Confidence/Self-Efficacy (1 Question)

Standard: Demographics (8 Questions)

Start of Block: Background

What is your current academic status?

- Current student
- Postdoc
- Recent graduate (but not currently a postdoc)

What degree(s) are you pursuing OR do you hold? (select one)

- MD
- MD/PhD
- PhD

Display This Question:

If What is your current academic status? = Postdoc

Or What is your current academic status? = Recent graduate (but not currently a postdoc)

| 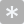 |
| --- |

What year did you complete your ${Q7/ChoiceGroup/SelectedChoices}?

________________________________________________________________

Display This Question:

If What is your current academic status? = Current student

What year of the program are you in?

- First year
- Second year
- Third year
- Fourth year

Display This Choice:

If What degree(s) are you pursuing OR do you hold? (select one) = MD/PhD

Or What degree(s) are you pursuing OR do you hold? (select one) = PhD

- Fifth year or beyond

Display This Question:

If What degree(s) are you pursuing OR do you hold? (select one) = MD

Or What degree(s) are you pursuing OR do you hold? (select one) = MD/PhD

For your MD, what institution are you enrolled in OR did you graduate from?

- Duke University
- North Carolina Central University
- UNC-Chapel Hill
- NC State University
- Other __________________________________________________

Display This Question:

If What degree(s) are you pursuing OR do you hold? (select one) = PhD

Or What degree(s) are you pursuing OR do you hold? (select one) = MD/PhD

For your PhD, what institution are you enrolled in OR did you graduate from?

- Duke University
- North Carolina Central University
- UNC-Chapel Hill
- NC State University
- Other __________________________________________________

Display This Question:

If What is your current academic status? = Postdoc

At which institution are you completing your postdoctoral work?

- Duke University
- North Carolina Central University
- UNC-Chapel Hill
- NC State University
- Other __________________________________________________

When did you first seriously consider pursuing a  ${Q7/ChoiceGroup/SelectedChoices}? (select one)

- Before undergraduate (K-12)
- 1st or 2nd year of undergraduate
- 3rd or 4th year of undergraduate
- After undergraduate
- Other __________________________________________________

Start of Block: Experience in UG/Preparation for admission

Now, we want to know a bit about your experience as an undergraduate.

What institution did you attend for your undergraduate education?

________________________________________________________________

Is this institution considered...

|  | Yes | No | Unsure |
| --- | --- | --- | --- |
| A historically black college or university (HBCU)? |  |  |  |
| A small liberal arts college? |  |  |  |
| A research intensive (R1) institution? |  |  |  |

In your experience, how competitive were students with each other at your undergraduate institution?

- Not at all competitive
- Slightly competitive
- Moderately competitive
- Very competitive
- Extremely competitive

Think about your effort academically as an undergraduate.
 
How hard did you have to work to perform well?

- I always had to work very hard to perform well academically. (1)
- (2)
- (3)
- (4)
- I never had to work hard to perform well academically (performing well academically was easy for me) (5)

How important were each of the following when deciding which graduate program(s) to apply to?

|  | Importance to decision | | | | | | |
| --- | --- | --- | --- | --- | --- | --- | --- |
|  | Not at all important (1) | 2 | 3 | Moderately important (4) | 5 | 6 | Absolutely critical (7) |
| Advice from my undergraduate professors |  |  |  |  |  |  |  |
| Familiarity with faculty at the program |  |  |  |  |  |  |  |
| Prior students from UG institution attended the program |  |  |  |  |  |  |  |
| Recruitment of students from UG institution by graduate program |  |  |  |  |  |  |  |
| High-ranking program (e.g., academic ranking, job placement) |  |  |  |  |  |  |  |
| Fit with your specific academic interests (e.g., your research interests) |  |  |  |  |  |  |  |
| Cost |  |  |  |  |  |  |  |
| Location |  |  |  |  |  |  |  |
| Other: |  |  |  |  |  |  |  |

Start of Block: Application

Now, we want to know about your experience preparing for and applying to your ${Q7/ChoiceGroup/SelectedChoices} program. 
 
If you applied to a ${Q7/ChoiceGroup/SelectedChoices} program more than once, please think about the most recent time you applied (i.e., just before you started your ${Q7/ChoiceGroup/SelectedChoices} program).

Based on your experience applying to graduate school: 


a) How **important** do you think each of the following skills/experiences are for a successful application? 


b) How would you **evaluate yourself** on each of those skills/experiences at the time of your application?

|  | a) Importance to successful application | | | | | | | b) Your strength when applying | | | | | | |
| --- | --- | --- | --- | --- | --- | --- | --- | --- | --- | --- | --- | --- | --- | --- |
|  | Extremely unimportant | Moderately unimportant | Slightly unimportant | Neither unimportant nor important | Slightly important | Moderately important | Extremely important | Extremely weak (novice) | Moderately weak | Slightly weak | Neither weak nor strong | Slightly strong | Moderately strong | Extremely strong (expert) |
| Knowing what is needed for an application |  |  |  |  |  |  |  |  |  |  |  |  |  |  |
| Completing applications |  |  |  |  |  |  |  |  |  |  |  |  |  |  |
| Research/lab experience |  |  |  |  |  |  |  |  |  |  |  |  |  |  |
| GRE/MCAT preparation |  |  |  |  |  |  |  |  |  |  |  |  |  |  |
| Reading academic/scientific literature |  |  |  |  |  |  |  |  |  |  |  |  |  |  |
| Basic laboratory competency |  |  |  |  |  |  |  |  |  |  |  |  |  |  |
| Quantitative skills (e.g., statistics, calculus) |  |  |  |  |  |  |  |  |  |  |  |  |  |  |
| Critical thinking |  |  |  |  |  |  |  |  |  |  |  |  |  |  |
| Writing and communication skills |  |  |  |  |  |  |  |  |  |  |  |  |  |  |
| Time management |  |  |  |  |  |  |  |  |  |  |  |  |  |  |
| Network connections (e.g., getting to know professors) |  |  |  |  |  |  |  |  |  |  |  |  |  |  |
| Good recommendation letter(s) |  |  |  |  |  |  |  |  |  |  |  |  |  |  |

Besides items above, are there other skills/experiences you think are critical for having a successful application? Please describe.

________________________________________________________________

Besides items above, do you think you had particular strengths or weaknesses that affected your competitiveness when applying? Please describe.

________________________________________________________________

When you first applied to a ${Q7/ChoiceGroup/SelectedChoices} program, how confident were you that you would be admitted to a program you wanted?

- Not at all confident
- Slightly confident
- Moderately confident
- Very confident
- Extremely confident

Did you complete a post-baccalaureate program?

- Yes
- No

Start of Block: Postbac

Which post-baccalaureate program did you complete?

________________________________________________________________

Why did you choose to complete a post-baccalaureate program? (select all that apply)

- Undergraduate faculty advice
- I thought I needed additional skills/experience
- Previously unsuccessful application to a graduate biomedical sciences program
- Other __________________________________________________

How much do you feel the program helped with gaining admission into your ${Q7/ChoiceGroup/SelectedChoices} program?

- Not at all helpful
- A little helpful
- Moderately helpful
- Very helpful
- Extremely helpful

Please explain.

________________________________________________________________

How useful was the post-baccalaureate program in improving each of the following?

|  | Usefulness | | | | | |
| --- | --- | --- | --- | --- | --- | --- |
|  | Not useful | Somewhat useful | Moderately useful | Very useful | Extremely useful | N/A; program did not address |
| Knowing what is needed for an application |  |  |  |  |  |  |
| Completing applications |  |  |  |  |  |  |
| Gaining research/lab experience |  |  |  |  |  |  |
| GRE/MCAT preparation |  |  |  |  |  |  |
| Reading academic/scientific literature |  |  |  |  |  |  |
| Basic laboratory competency |  |  |  |  |  |  |
| Quantitative skills (e.g., statistics, calculus) |  |  |  |  |  |  |
| Critical thinking |  |  |  |  |  |  |
| Writing and communication skills |  |  |  |  |  |  |
| Time management |  |  |  |  |  |  |
| Network connections (e.g., getting to know professors) |  |  |  |  |  |  |
| Good recommendation letter(s) |  |  |  |  |  |  |
| Other |  |  |  |  |  |  |

What was most beneficial about the post-baccalaureate program, if anything?

________________________________________________________________

What could have been improved about the post-baccalaureate program, if anything?

________________________________________________________________

Start of Block: Experience & needs in grad program

Based on your experience in graduate school:


a) How **important** do you think each of the following skills/experiences are/were for success in your program? 


b) How **challenging** are/were these skills/experiences for you?

|  | a) Importance to success | | | | | | | b) How challenging | | | | | | |
| --- | --- | --- | --- | --- | --- | --- | --- | --- | --- | --- | --- | --- | --- | --- |
|  | Extremely unimportant | Moderately unimportant | Slightly unimportant | Neither | Slightly important | Moderately important | Extremely important | Extremely challenging | Moderately challenging | Slightly challenging | Neither challenging nor easy | Slightly easy | Moderately easy | Extremely easy |
| Research/lab experience |  |  |  |  |  |  |  |  |  |  |  |  |  |  |
| Reading academic/scientific literature |  |  |  |  |  |  |  |  |  |  |  |  |  |  |
| Basic laboratory competency |  |  |  |  |  |  |  |  |  |  |  |  |  |  |
| Quantitative skills (e.g., statistics, calculus) |  |  |  |  |  |  |  |  |  |  |  |  |  |  |
| Critical thinking |  |  |  |  |  |  |  |  |  |  |  |  |  |  |
| Writing and communication skills |  |  |  |  |  |  |  |  |  |  |  |  |  |  |
| Time management |  |  |  |  |  |  |  |  |  |  |  |  |  |  |
| Network connections (e.g., getting to know professors) |  |  |  |  |  |  |  |  |  |  |  |  |  |  |
| Other |  |  |  |  |  |  |  |  |  |  |  |  |  |  |

In addition to the skills/experiences listed above, how challenging were each of the following aspects of your graduate experience?

|  | How challenging | | | | | | |
| --- | --- | --- | --- | --- | --- | --- | --- |
|  | Extremely challenging | Moderately Challenging | Slightly challenging | Neither challenging nor easy | Slightly easy | Moderately easy | Extremely easy |
| Competitive culture of my graduate program |  |  |  |  |  |  |  |
| Feelings of isolation |  |  |  |  |  |  |  |
| Imposter syndrome (i.e., feelings of inadequacy) |  |  |  |  |  |  |  |

A “mentor” is someone who provides guidance, assistance, and encouragement on professional and academic issues. A mentor is more than an academic advisor; they are someone you turn to for guidance and assistance beyond selecting classes or meeting academic requirements. 
 
During your graduate program, do/did you have a mentor that you go/went to for support?

- Yes, I participate in a formal mentor program
- Yes, I have an informal mentor
- No
- I'm not sure

Display This Question:

If A “mentor” is someone who provides guidance, assistance, and encouragement on professional and ac... = Yes, I participate in a formal mentor program

Or A “mentor” is someone who provides guidance, assistance, and encouragement on professional and ac... = Yes, I have an informal mentor

How much has mentorship helped you feel like you can/did succeed in your program?

- Not at all
- Somewhat
- Moderately
- Very
- Extremely

Display This Question:

If A “mentor” is someone who provides guidance, assistance, and encouragement on professional and ac... = Yes, I participate in a formal mentor program

And A “mentor” is someone who provides guidance, assistance, and encouragement on professional and ac... = Yes, I have an informal mentor

What about mentorship during your graduate program is/was helpful, if anything?

________________________________________________________________

Display This Question:

If A “mentor” is someone who provides guidance, assistance, and encouragement on professional and ac... = Yes, I participate in a formal mentor program

And A “mentor” is someone who provides guidance, assistance, and encouragement on professional and ac... = Yes, I have an informal mentor

What about mentorship during your graduate program is/was unhelpful, if anything?

________________________________________________________________

What is the top piece of advice you would give to an incoming graduate student in the biomedical sciences?

________________________________________________________________

Start of Block: Confidence/Self-Efficacy

Please share how much you agree/disagree with the following statements about your work in your ${Q7/ChoiceGroup/SelectedChoices} program.

|  | Strongly disagree | Somewhat disagree | Neither agree nor disagree | Somewhat agree | Strongly agree |
| --- | --- | --- | --- | --- | --- |
| I will be able to achieve most of the goals that I have set for myself. |  |  |  |  |  |
| When facing difficult tasks, I am certain that I will accomplish them. |  |  |  |  |  |
| In general, I think that I can obtain outcomes that are important to me. |  |  |  |  |  |
| I believe I can succeed at most any endeavor to which I set my mind. |  |  |  |  |  |
| I will be able to successfully overcome many challenges. |  |  |  |  |  |
| I am confident that I can perform effectively on many different tasks. |  |  |  |  |  |
| Compared to other people, I can do most tasks very well. |  |  |  |  |  |
| Even when things are tough, I can perform quite well. |  |  |  |  |  |

Start of Block: Demographics

What is your racial and ethnic identity? (check all that apply)
*Note: this list is adapted from NIH guidelines and we recognize the potential challenges of these racial identity categorizations.*

- Asian/South Asian/Asian American
- Black/African American
- Hispanic/Latino
- Native American/Alaska Native
- Native Hawaiian
- Other Pacific Islander
- Middle Eastern
- White/European American

What is your gender identity?

- Female
- Male
- Non-binary/third gender
- Self-identify: __________________________________________________
- Prefer not to say

| 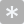 |
| --- |

What is your age in years?

________________________________________________________________

Were you born outside of the United States?

- Yes
- No

Were one or either of your parents/guardians born outside of the United States?

- Yes
- No
- Unknown/not sure

What is the highest level of education achieved by your parent(s)/guardian(s)?

- Less than a high school diploma
- High school degree or equivalent (e.g. GED)
- Some college, no degree
- Associate degree (e.g. AA, AS)
- Bachelor’s degree (e.g. BA, BS)
- Master’s degree (e.g. MA, MS, MEd)
- Professional degree (e.g. MD, DDS, DVM)
- Doctorate (e.g. PhD, EdD)
- Unknown/not sure

The Americans with Disabilities Act defines a person with a disability as a person who has a physical or mental impairment that substantially limits one or more major life activity. Have you ever had or are you currently considered to have a disability?

- Yes
- No

Check all of the following that apply or have applied to you:

- Were homeless
- Were in the foster care system
- Were eligible for the Federal Free and Reduced Lunch Program for two or more years
- Were or currently are eligible for Federal Pell grants
- Received support from the Special Supplemental Nutrition Program for Women, Infants and Children (WIC) as a parent or child
- Grew up in a rural area
